# Psoralen synergies with zinc implants to promote bone repair by regulating ZIP4 in rats with bone defect

**DOI:** 10.1186/s40824-023-00472-w

**Published:** 2023-12-10

**Authors:** Meijing Liu, Junlong Tan, Shuang Li, Chaoyang Sun, Xiangning Liu, Hongtao Yang, Xiaogang Wang

**Affiliations:** 1 The First Affiliated Hospital of Jinan University, Clinical Research Platform for Interdiscipline of Stomatology, School of Stomatology, Jinan University, Guangzhou, 510630 PR China; 2 Key Laboratory of Big Data-Based Precision Medicine, School of Engineering Medicine, Beihang University, Beijing, 100191 PR China

**Keywords:** Zinc implants, ZIP4, Cytotoxicity, Psoralen, Osteogenesis, Bone repair

## Abstract

**Background:**

The regulation of dose-dependent biological effects induced by biodegradation is a challenge for the production of biodegradable bone-substitute materials, especially biodegradable zinc (Zn) -based materials. Cytotoxicity caused by excess local Zn ions (Zn^2+^) from degradation is one of the factors limiting the wide application of Zn implants. Given that previous studies have revealed that delayed degradation of Zn materials by surface modification does not reduce cytotoxicity; in the present study, we explore whether preventing the entry of excess Zn^2+^ into cells may can reduce local Zn toxicity by applying Psoralen (PRL) to Zn implants and assessing its ability to regulate intracellular Zn^2+^ concentrations.

**Methods:**

The effects of different concentrations of Zn^2+^ on cellular activity and cytotoxicity were investigated; briefly, we identified natural compounds that regulate Zn transporters, thereby regulating the concentrations of intracellular Zn^2+^, and applied them to Zn materials. Of these materials, PRL, a natural, tricyclic, coumarin-like aromatic compound that promotes the proliferation and differentiation of osteoblasts and enhances osteogenic activity, was loaded onto the surface of a Zn material using peptides and chitosan (CS), and the surface characteristics, electrochemical properties, and activity of the modified Zn material were evaluated. In addition, the ability of Zn + CS/pPRL implants to promote bone formation and accelerate large-scale bone defect repairs was assessed both in vitro and in vivo.

**Results:**

We determined that 180 µM Zn^2+^ significantly induced pre-osteoblast cytotoxicity, and a 23-fold increase in Zrt- and Irt-like protein 4 (ZIP4) expression. We also found that PRL dynamically regulates the expression of ZIP4 in response to Zn^2+^ concentration. To address the problem of cytotoxicity caused by excessive Zn^2+^ in local Zn implants, PRL was loaded onto the surface of Zn implants in vivo using peptides and CS, which dynamically regulated ZIP4 levels, maintained the balance of intracellular Zn^2+^ concentrations, and enhanced the osteogenic activity of Zn implants.

**Conclusions:**

This study reveals the importance of Zn^2+^ concentration when using Zn materials to promote bone formation and introduces a natural active ingredient, PRL, that can regulate intracellular Zn^2+^ levels, and thus may be clinically applicable to Zn implants for the treatment of critical bone defects.

**Graphical Abstract:**

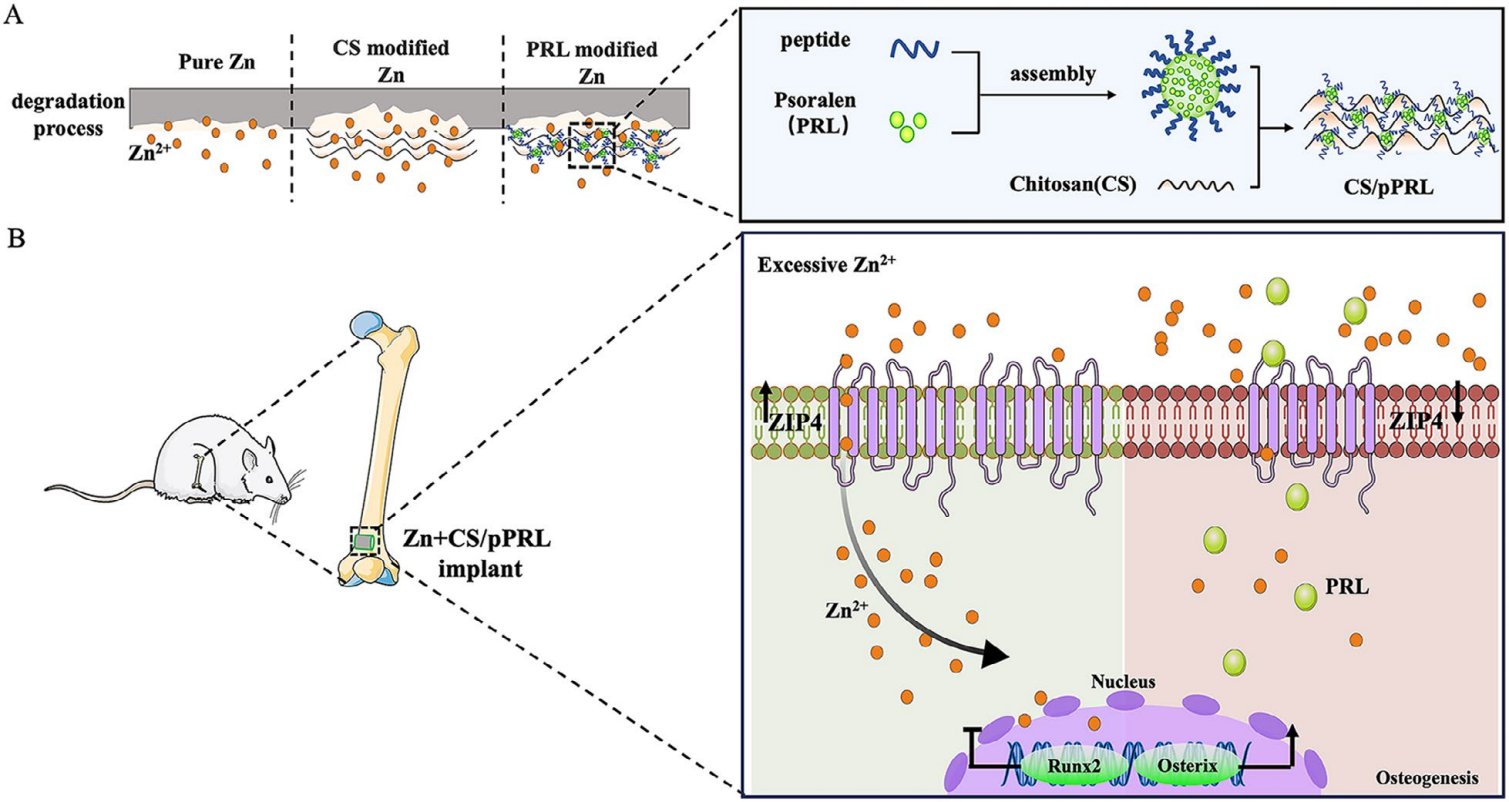

**Supplementary Information:**

The online version contains supplementary material available at 10.1186/s40824-023-00472-w.

## **Introduction**

Bone defects are a serious problem in clinical orthopedics. Although the success rate of bone fracture healing by forming a cartilaginous callus is generally high [1–3], bone defects larger than 3 cm cannot form a callus, which increases complication rates, and can result in a significant clinical burden [4]. Current treatments for large bone defects depend on external medical implants such as autografts, allografts, and artificial bone scaffolds [5–7]. However, except for autogenous bone, most of the materials used in such implants do not exhibit strong biocompatibility, bone formation, bone conduction, or bone induction. Thus, the search for an ideal bone repair material remains a popular research topic.

Zinc (Zn) and its alloys, as promising medical biodegradable metal implants, show many superior properties to other materials, including higher mechanical strength and an elastic modulus similar to that of bone tissue [8, 9]. Moreover, human bone tissue storage represents 85% of Zn, which plays a vital role in bone formation and mineralization [10, 11]. Studies have shown that, during bone formation, Zn promotes proliferation, differentiation, and collagen synthesis in osteoblasts [12–15]; however, in vivo Zn content has biphasic biological activity. Specifically, a lack or excess of Zn causes physiological or pathological changes that can lead to a variety of diseases. For example, Zn overdose is known to induce cell death [16], and previous studies have associated Zn toxicity with liver and kidney damage in vivo [17, 18]. The locally high concentration of Zn^2+^ in Zn implants may be the reason for the unsatisfactory effect of Zn in promoting bone formation. Addressing the toxicity in cells adjacent to biodegradable Zn scaffolds remains a challenge.

Organs with abundant Zn content in the human body include the eyes, hair, bones, and skin [19]. The transport of Zn in and out of cells is regulated by Zn transporters, which are carrier proteins present in the plasma membrane of cells and various organelles that act as regulatory gates for Zn transport [20, 21]. The zinc-iron-related transporter protein (ZIP) family consists of 14 members that are responsible for zinc uptake from the extracellular milieu or intracellular vesicles into the cytoplasm, thereby increasing the content of free zinc in the cytoplasm [21, 22]. ZIP4, also known as solute carrier family 39 member 4 (SLC39A4), has been shown to regulate concentrations of intracellular Zn^2+^, including in pancreatic cancer [23, 24] and metabolic diseases [25, 26]. Studies have also shown that the level of Zn^2+^ in the small intestine, liver, pancreas, and other organs of intestinal ZIP4 knockout mice decreased sharply over the course of the disease, causing bone mass loss [27]. In addition, ZIP4 mutant mice exhibit abnormal skeleton morphology [28]. Paradoxically, ZIP4 knockdown in human pancreatic cancer cells increased bone tissue mineral density in an orthotopic xenograft mouse model [29]. Overall, ZIP4 plays an important role in the regulation of Zn^2+^ levels and treatment of bone-related diseases.

Psoralen (PRL) is a furanocoumarin compound extracted from *Psoralea corylifolia Linn*. that contains anti-inflammatory, anti-osteoporotic, and neuroprotective properties. Studies have shown that PRL can activate osteoblasts through ERK and BMP signaling pathways, induce osteogenic differentiation, increase the number of osteoblasts in the fracture callus, and accelerate fracture healing [30, 31]. Most importantly, PRL in combination with Zn, has reportedly enhance osteoblast proliferation and differentiation in rats [32]. In this study, we assessed how the dual characteristics of PRL affect ZIP4 regulation of Zn^2+^ concentrations in vivo, resolve the issues of cytotoxicity and biocompatibility of Zn implants, and synergistically promote bone formation and accelerate bone repair in rats with bone defects.

## Materials and methods

### Materials

PRL (C_11_H_6_O_3_, molecular weight (Mw) = 186.163, 98.29% purity) was purchased from Chengdu Biochem Pure Biotechnology Co. LTD (Sichuan, China), peptides (Palmitic acid-VVVV-DDSESSE) were purchased from Hangzhou Zhuantai Biotechnology Co., LTD (Hangzhou, China), and Zn rods (99.99%, metal-based) were purchased from Alfa Aesar (Shanghai, China).

### Construction of Zn + CS/pPRL implants

A solution of pPRL nanoparticles (1 mg/mL) was prepared in psoralen (PRL) and the peptide was prepared (palmitic acid-VVVV-DDSESSE) in phosphate-buffered saline (PBS). A 1 mg/mL chitosan (CS) solution was prepared and added to the pPRL (v:v = 1:1) solution. A zinc rod (3 mm × 4 mm) was soaked in the CS/pPRL coating solution for 10 min, dried overnight in a biological drying oven at 37 °C, and stored at 25 °C and 40–60% humidity.

### Characterization of pPRL nanoparticles and Zn + CS/pPRL implants

A volume of 10 µL of the peptide or pPRL nanoparticle solutions was deposited on a copper grid coated with a carbon film and negatively stained with 2.5% phosphotungstic acid. Transmission electron microscopy (TEM) images were acquired using a JEM-2100 microscope (JEOL, Japan). The surface microstructures of pure Zn or Zn + CS/pPRL implants were examined on gold-coated samples using scanning electron microscopy (SEM, Hitachi S-4800, Japan).

### Electrochemical tests

The electrochemical behaviors of pure Zn, Zn + CS, and Zn + CS/pPRL were analyzed using an electrochemical workstation at 37 °C in simulated body fluid (SBF) solution (Autolab, Metrohm, Switzerland) containing 8.035 g/L NaCl, 0.355 g/L NaHCO_3_, 0.225 g/L KCl, 0.231 g/L K_2_HPO_4_·3H_2_O, 0.311 g/L MgCl_2_·6H_2_O, 0.292 g/L CaCl_2_, 0.072 g/L Na_2_SO_4_, and 6.118 g/L Tris. The pH value of the SBF solution was adjusted to 7.4 with HCl solution (1 M) at 37 °C. A platinum mesh and saturated calomel electrode (SCE) were used as the counter and reference electrodes, respectively. The open-circuit potential (OCP) for each sample with a test area of 0.2826 cm^2^ was monitored for 3600 s. Potentiodynamic polarization (PDP) curves were measured at a scanning rate of 1 mV/s, and the corrosion potential (E_corr_) and corrosion current density (i_corr_) of the samples were calculated by extrapolating the polarization curve [33].

### HPLC analysis of PRL concentrations in Zn implant extracts

Zn + CS/pPRL implant extracts were prepared in PBS for 24 h. The ratio of the Zn surface area (cm^2^) to PBS volume (mL) was set to 1.25 cm^2^/mL for all samples. PRL concentrations in the Zn implant extracts were analyzed using an Agilent 1200 HPLC system (Agilent Technologies, Santa Clara, CA, USA), and compound separations was carried out using an Agilent C_18_ column (5 μm, 250 mm × 4.6 mm) at 25 °C. Analysis was performed using a mobile phase consisting of a 20 µL injection of acetonitrile/water (30:70, v/v) at a flow rate of 1.0 mL/min. All samples were detected at a wavelength of 245 nm. Linear calibration plots for the proposed method were obtained over corresponding concentration ranges of 0.5–25.0 and at 0.5, 1.0, 2.5, 5.0, 10.0 and 25.0 µg/mL for PRL. Each solution was prepared in triplicates.

### Molecular docking

The chemical structure of PRL (PubChem CID: 6199) was obtained from the PubChem database (https://pubchem.ncbi.nlm.nih.gov) and prepared using the Autodock-4.2.6 software. The three-dimensional structure of ZIP4 (PDB code: 4X 82), with a resolution of 2.76 Å, was obtained from the RCSB PDB (http://www.rcsb.org). The co-crystallized structure was prepared using Autodock-4.2.6. All water molecules and ligands from the protein structures were removed, and hydrogen atoms were added. The prepared structure of the ZIP4 protein was submitted to Autodock-4.2.6 for molecular docking analysis. The docking of molecules and proteins was performed using Autodock-4.2.6, then the docking results were analyzed. A pose with the minimum binding energy score was selected, further analyzed, and visualized using PyMOL-2.4.1.

### Cell culture

The osteoblast precursor cell line (MC3T3-E1) was purchased from the Cell Bank of the Shanghai Institute (Shanghai, China). The MC3T3-E1 were cultured in Minimum Essential Medium α (α-MEM, Gibco) with 10% fetal bovine serum (FBS, Gibco) and 1% antibiotic-antimycotic (100 U/mL penicillin and 0.1 mg/mL streptomycin) at 37 ℃ in a humid, 5% CO_2_ atmosphere.

### Cell survival analysis

MC3T3-E1 cells were seeded in 96-well plates (5 × 10^4^ cells/well) and cultured for 24 h. The fresh culture media containing different concentrations of Zn^2+^ (360, 180, 90, 45, 22.5, and 0 µM) or PRL (0, 1, 2, 5, 10, 20, 50, 100, and 200 µM) were replaced to and cultured for 48 h. Then, 10 µL of Cell Counting Kit-8 (CCK-8, Dojindo, Japan) solution was added into each well, and the solution was incubated for 1 h. The absorbance was measured at 450 nm using a microplate reader (Bio-RAD680). The cell survival rate was calculated according to the formula described in the manufacturer’s instructions.

### Terminal dUTP nick-end labeling (TUNEL) assay

Zinc ion-induced DNA damage in preosteoblasts was detected using a TUNEL apoptosis assay kit (C1089, Beyotime, China). The MC3T3-E1 cells (2.5 × 10^5^ cells/well) were precultured in a 6-well plate for 24 h, after which the fresh culture media containing different concentrations of Zn^2+^ (180, 90, 45, 22.5, 0 µM) were replaced, then cultured for 48 h. Cells were then fixed in 4% paraformaldehyde (PFA) for 20 min, rinsed with PBS and incubated with 0.3% Triton X-100 for 5 min at 4 °C. The TUNEL test solution was added and incubated for 60 min at 37 °C in the dark. After washing, the cells were incubated with 4,6-diamidino-2-phenylindole (DAPI; Sigma) for 10 min, followed by exhaustive washing in PBS. Apoptotic cells were visualized under a confocal microscope (Leica).

### Gene silencing

MC3T3-E1 cells were seeded in plates for 24 h. Cells were transfected with 50 µM of siRNA targeting mouse Zip4 mRNA and siRNA control using Lipofectamine 3000 for 24 h. Then, cells were treated with the fresh culture media containing Zn^2+^ (0, 180µM) and DMSO, PRL (1, 2, 5µM) for 24 h. Finally, cells were used for CCK-8 or TUNEL assay, as described.

### Alizarin red staining

Extracts were prepared by using culture medium (surface area to medium ratio of 1.25 mL/cm^2^) at 37 °C in a humidified 5% CO_2_ atmosphere for 24 h. Osteogenic differentiation of pre-osteoblasts was assessed using Alizarin Red Staining (Solarbio, China). Cells (2.5 × 10^5^ cells/well) were precultured in a 6-well plate for 24 h. Then, fresh culture medium containing 25% extracts or no extracts (control) was added every 2 d. The cells were cultured for 21 d, rinsed with PBS, and fixed in 4% paraformaldehyde (PFA) for 20 min. After fixation, the cells were washed with deionized water. Alizarin red solution (40 mM) was added, and the cells were incubated at room temperature for 20 min and then rinsed with deionized water. Images were acquired using a light microscope (Leica).

### ALP staining

Differentiation of preosteoblasts was measured using alkaline phosphatase (ALP) staining (Solarbio, Beijing, China). MC3T3-E1 cells (2.5 × 10^5^ cells/well) were pre-cultured in a 6-well plate for 24 h. Then, fresh culture medium containing 25% extracts or no extracts (control) was added every 2 d. The cells were cultured for 14 days, rinsed with PBS, and fixed in 4% paraformaldehyde (PFA) for 30 min. After fixing, cells were washed with PBS three times and incubated with NBT (nitro-blue tetrazolium chloride) and BCIP (5-bromo-4-chloro-3’-indolyphosphate p-toluidine salt) solution (Solarbio, China) for 30 min. Images were acquired using a light microscope (Leica).

### Actin staining of the cytoskeleton

MC3T3-E1 cells (1 × 10^5^) were seeded into a 24-well culture plate containing Zn sheets (1 cm diameter) with CS or CS/pPRL, and 1 mL of the medium was used to culture the cells for 24 h. Similarly, MC3T3-E1 (2.5 × 10^5^) were seeded in glass bottom cell culture dishes (#801,001, NEST, China) and cultured for 24 h. The fresh culture medium containing different concentrations of Zn^2+^ (180, 90, 45, 22.5, or 0 µM) or 25% extracts were added to cell plates and cultured for 48 h.

The cells were then fixed with 4% paraformaldehyde for 10 min at room temperature, permeabilized with 0.1% TritonX-100 in PBS for 20 min, and blocked with 5% bovine serum albumin (BSA) at room temperature for 30 min. Subsequently, the cells were stained with FITC-phalloidin (CA1620, Solarbio Life Sciences, Beijing, China) for 2 h at room temperature in the dark. After washing, the cells were incubated with DAPI solution for 10 min, followed by exhaustive washing with PBS. F-actin distribution was observed under a confocal microscope (Leica).

### Detection of cellular Zn^2+^ content by zinc probe

Cell samples and bone sections were prepared. Dissolve the FluoZin^TM^-3, AM (F24195, Invitrogen) in water containing DMSO, Pluronic^@^ F-127 (P6867, Invitrogen) according to the manufacturer’s instructions. The samples were then incubated in the FluoZin^TM^-3, AM aqueous solution for 30 min at 37 °C. After washing, the cells were incubated with 4,6-diamidino-2-phenylindole (DAPI; Sigma) for 10 min, followed by exhaustive washing in PBS. The fluorescence intensity was observed using a confocal microscope (Leica).

### Animals and experimental design

All animal experiments were approved by the Institutional Animal Care and Use Committee of the Beihang University. A total of 24 adult male Wistar rats at 12-week-old were purchased from Beijing Huafukang Biotechnology Co., LTD (SCXK(JING)2019-0008) for modified zinc implantation surgery. Animals had ad libitum access to water and food during the experiment. Briefly, rats were anesthetized, and homeostasis was maintained with isoflurane in oxygen. The skin of the left hind limb of the rat was prepared, and the knee joint was fixed. Next, a longitudinal incision was made approximately 15 mm lateral to the patellar ligament, and the lateral femoral condyle was exposed via blunt dissection. A cylindrical bone defect, 3 mm in diameter and 4 mm in depth, was implanted using a 3 mm drill. The defect site was washed with saline, the Zn scaffold was implanted, and the incision was closed layer-by-layer. The rats were divided into control (bone defect model control group), Zn (pure zinc scaffolds), Zn + CS (CS-coated zinc scaffolds), and Zn + CS/pPRL (CS-loaded pPRL nanoparticles of zinc scaffolds) groups, with six rats per group. At 8 weeks after implantation, blood was collected from anesthetized rats, and then the rats were sacrificed, femurs and other tissues were collected.

### Micro-CT analysis

The femur with the zinc implant was scanned using a high-resolution micro-CT (SkyScan 1176, Brucker) scanner at an isometric solution of 15 μm (85 kV and 200 µA) to evaluate the in vivo osteointegration of the implants. The regions of interest (ROIs) were determined by size matching with the implants (D:5.0 mm, H:5.0 mm). The bone tissue volume/total tissue volume (BV/TV) ratio was then calculated.

### Analysis of serum biomarkers of bone formation

Blood was centrifuged at 1000 × g for 10 min at 4℃. The levels of serum bone formation biomarkers, namely, bone alkaline phosphatase (BALP, CSB-E11865r) and procollagen type I N-terminal propeptide (PINP, CSB-E12774r), were measured using an ELISA kit (CUSABIO, Wuhan, China) following the manufacturer’s instructions.

### Fluorescent labeling of newly formed bone

To evaluate new bone growth rate, calcein and xylenol orange were used to mark the new bone tissue. In brief, calcein (15 mg/kg, #B9140, Solarbio, Beijing, China) was injected into the rats via intraperitoneal injection, and after 2 weeks xylenol orange (30 mg/kg, #C7600, Solarbio, Beijing, China) was injected. Finally, the rats were sacrificed and the femurs were collected, fixed in 4% PFA solution, and embedded in light-cured resin. A micro section and grinding system was used to cut the bone into 4 μm thick sections. Bone sections were rinsed and incubated with 4,6-diamidino-2-phenylindole (DAPI, Sigma) for 15 min, followed by exhaustive washing in phosphate-buffered saline (PBS). Images were captured using a fluorescence microscope (Leica).

### Analysis of Zn^2+^ concentration in serum

Blood was centrifuged at 1000 × g for 10 min at 4℃. The concentrations of zinc-containing compounds in the serum were measured using ICP-MS (Nexion 300, USA).

### Quantitative real-time PCR analysis

Total RNA was isolated from bone tissues or cells using TRIzol reagent (Invitrogen, USA). The cDNA was synthesized using PrimeScript™ RT Master Mix Kit (Takara, Tokyo, Japan), and TB Green® Premix Ex Taq™ II (Takara, Tokyo, Japan) in a CFX96™ Real-Time System (Bio-Rad Laboratories, Hercules, CA) according to the manufacturer’s protocol. The primer sequences used for gene amplification are listed in Table 1. All experiments were performed in triplicate and analyzed using the 2^−∆∆Ct^ method. For standardization and quantification, *Gapdh* mRNA was amplified simultaneously.

**Table 1:** The gene primer sequences for qPCR

	Primer	Sequences (5’-3’)
Mouse Zip1	Zip1-F	CTGGACCTGCTGCCTGACTAC
	Zip1-R	TGCCCAGTAGAGCCCTTGTC
Mouse Zip2	Zip2-F	ACTGGGCTGTGGCCTTACTC
	Zip2-R	CTTCCTGTACTATTCTGTTCCACG
Mouse Zip3	Zip3-F	TGCCTGCAGTGAGGGACAAG
	Zip3-R	ATCTGAGCCGGCGTTGAAGG
Mouse Zip4	Zip4-F	GGGCCGTGTGAAAAGTGTC
	Zip4-R	TTAAGGTAGAGGGCAGCAGC
Mouse Zip5	Zip5-F	CCTAGCAGTCTTCTGCCACGAG
	Zip5-R	GAAGCAGGGTTGGCAGCATATC
Mouse Zip6	Zip6-F	GAGAGCAAGAAGCAGCTGTCC
	Zip6-R	ATCGTCGGCTGCTGGGAATC
Mouse Zip7	Zip7-F	GGGCCACAGTGCTGATCTCC
	Zip7-R	GGTGAGAATGAGGTTCCAAGGC
Mouse Zip8	Zip8-F	TCGCCTTCAGTGAGGATGTGC
	Zip8-R	TGTAAGATCGCGGGGCAGATG
Mouse Zip9	Zip9-F	TGCTGGTCTGGAGCGGAATC
	Zip9-R	GTGGCTGTGCCCCATTCCA
Mouse Zip10	Zip10-F	TGGTGATCATGGGTGACGGC
	Zip10-R	TGCTTTACGGTCATGCCTGC
Mouse Zip11	Zip11-F	CCGGATAGGTAGCACTGGGC
	Zip11-R	CGCCAACAGCAAGACCCTCT
Mouse Zip12	Zip12-F	CCTGGTTGGAGATAGCCTGCAC
	Zip12-R	AAGGCCGTCAGGGCACTTAG
Mouse Zip13	Zip13-F	GGGCTCTCTCATGGTTGGGC
	Zip13-R	GGCGGGGGTGATGTTACAGG
Mouse Zip14	Zip14-F	GCGTTTTTCAGCCGTGTGCT
	Zip14-R	TATGCCCGTGATGGTGCTCG
Mouse Runx2	Runx2-F	CGAACAGAGCAACATCTCC
	Runx2-R	GTCAGTGCCTTTCCTTGG
Mouse Alpl	Alpl-F	TGCTACCTGCCTCACTTCC
	Alpl-R	GAGAGAAACCCACCCTGCT
Mouse Osterix	Osterix-F	GGCCTTTCGTCTGCAACT
	Osterix-R	TGCTCAAGTGGTCGCTTC
Mouse Gapdh	Gapdh-F	TGTGTCCGTCGTGGATCTGA
	Gapdh-R	TTGCTGTTGAAGTCGCAGGAG
Rat ZIP4	ZIP4-F	ATGAGCTGCCTCACGAACTT
	ZIP4-R	CTGCTAGAGCCACGTAGAGG
Rat RUNX2	RUNX2-F	GACTGTGGTTACCGTCATGGC
	RUNX2-R	ACTTGGTTTTTCATAACAGCGGA
Rat ALPL	ALPL-F	CCAACTCTTTTGTGCCAGAGA
	ALPL-R	GGCTACATTGGTGTTGAGCTTTT
Rat OSTERIX	OSTERIX-F	GGAAAGGAGGCACAAAGAAGC
	OSTERIX-R	CCCCTTAGGCACTAGGAGC
Rat GAPDH	GAPDH-F	CAAGAAGGTGGTGAAGCAG
	GAPDH-R	CAAAGGTGGAAGAATGGG

### H&E staining

Femurs were collected, fixed in 4% paraformaldehyde for 48 h, and decalcified with an EDTA decalcifying solution (pH = 7.4, #E1171, Solarbio, China) for 42 days, at which point the samples were dehydrated and embedded in paraffin. We prepared 4 μm thick coronal-oriented sections of the femur for hematoxylin and eosin (H&E) staining.

For liver, kidney, heart, and spleen sections, tissues were collected from euthanized rats and fixed with 4% paraformaldehyde for 48 h. Then, the tissue was dehydrated and embedded in paraffin, cut into 4-µm-thick slices, stained with H&E staining, and observed under a light microscope (Olympus, Japan).

### TRAP/ALP staining

TRAP and ALP staining were performed using the TRAP/ALP kit (#294-67001, Wako, Japan), according to the manufacturer’s instructions, to evaluate osteoclast and osteoblast formation. TRAP-positive cells stained red were considered osteoclasts and ALP-positive cells stained brown were considered activated osteoblasts or chondrocytes, as observed under a light microscope (Olympus, Japan).

### Statistical analysis

All results are expressed as the mean ± S.D. Numerical data were evaluated using GraphPad Prism Version 6.0, (GraphPad Software, La Jolla, CA, USA). The statistical significance of multiple comparisons was analyzed using one-way ANOVA, followed by the LSD test for homogeneity of variance. Dunnett’s method was used for variance differences. The difference between the two groups was analyzed using a Student’s unpaired t-test. *p* < 0.05 was considered statistically significant for all tests.

## Results

### Cytotoxicity caused by Zn^2+^ Overdose was associated with ZIP4

**Fig. 1:**
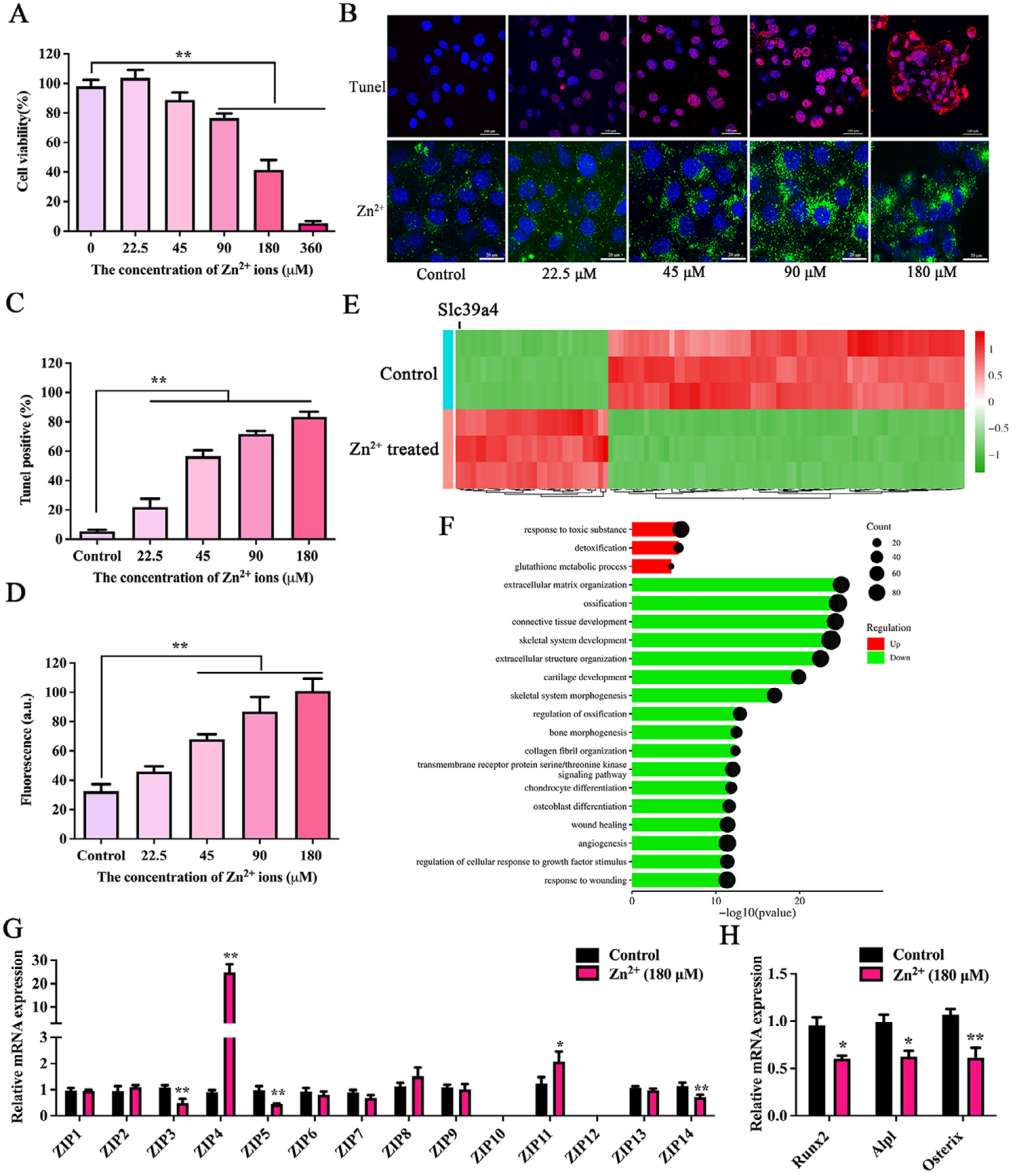
Analysis of the mechanism of cytotoxicity induced by excessive Zn^2+^. (**A**) MC3T3-E1 cells were incubated with cell medium containing different concentrations of Zn^2+^ for 24 h, and cell counting kit-8 (CCK-8) assay was used to detect cell viability. (**B**) Top: apoptotic cells were detected using a Terminal dUTP nick-end labeling (TUNEL) assay. Bottom: intracellular Zn^2+^ concentrations in MC3T3-E1 were detected using a Zn^2+^ fluorescence probe (FluoZin-3, AM). (**C**) The quantification analysis of positive TUNEL cells. (**D**) The fluorescence quantification analysis of intracellular Zn^2+^ concentrations. (**E**) RNA transcriptome sequencing analysis of MC3T3-E1 cells treated with excessive Zn^2+^ (180 µM) or control (n = 3). Heatmap of top-100 highly variable genes. (**F**) GO enrichment analysis results. (**G–H**) The expression Zip1-Zip14 mRNA and genes associated with osteogenesis (Runx2, Alpl, Osterix mRNA) in control cells or MC3T3-E1 cells treated with excessive Zn^2+^ were detected using RT-qPCR (n = 3). Bars represent mean ± S.D. *p < 0.05; **p < 0.01

Studies have shown that Zn^2+^ release creates toxicity in Zn implants [34]; however, the mechanism of cytotoxicity is unclear. In this study, cytotoxicity was evaluated using different assays based on cell viability and DNA injury at different concentrations of Zn^2+^. Results from cell counting experiments showed that the survival rate of MC3T3-E1 cells was approximately 5% when cultured in α-MEM medium containing Zn^2+^ (360 µM) (Fig. 1A). Our experimental results showed that, with an increase in Zn^2+^ concentration in cells, the proportion of DNA damage also increased (Fig. 1B-D). To elucidate the molecular mechanisms of Zn^2+^ cytotoxicity in MC3T3-E1 cells, we performed transcriptomic profiling using RNA sequencing. We focused on the top 100 differentially expressed genes (DEGs), those characterized by a large fold change and highly significant p-values. Of these, Zip4 (Slc39a4), a gene involved in Zn^2+^ transport, showed significantly increased expression (Fig. 1E). In addition, according to Gene Ontology (GO) function annotation, we found that excess Zn^2+^ prominently upregulated the transcriptional levels involved in the response to toxic substances and detoxification signaling pathways and downregulated the transcriptional levels involved in extracellular matrix organization, ossification, and osteoblast differentiation signaling pathways (Fig. 1F). These results suggest that excessive intracellular Zn^2+^ led to cytotoxicity and inhibition of bone formation. Next, regarding the expression of members 1 to 14 of the Zip family and osteogenesis-related genes after intervention with excessive Zn^2+^, we found a significant increase in the expression of Zip4 transporters when intracellular Zn^2+^ was excessive, but lower expressions of Runx2, Alpl, and Osterix mRNA (Fig. 1G-H). In addition, we observed that in the presence of excessive Zn^2+^, silencing ZIP4 expression in MC3T3-E1 resulted in increased the survival of cell and reduced the proportion of DNA damage (Fig. S1). Therefore, cytotoxicity caused by excessive local release of Zn^2+^ can be resolved by regulating ZIP4 expression.

### Psoralen regulates ZIP4 expression according to Zn^2+^ concentration

**Fig. 2:**
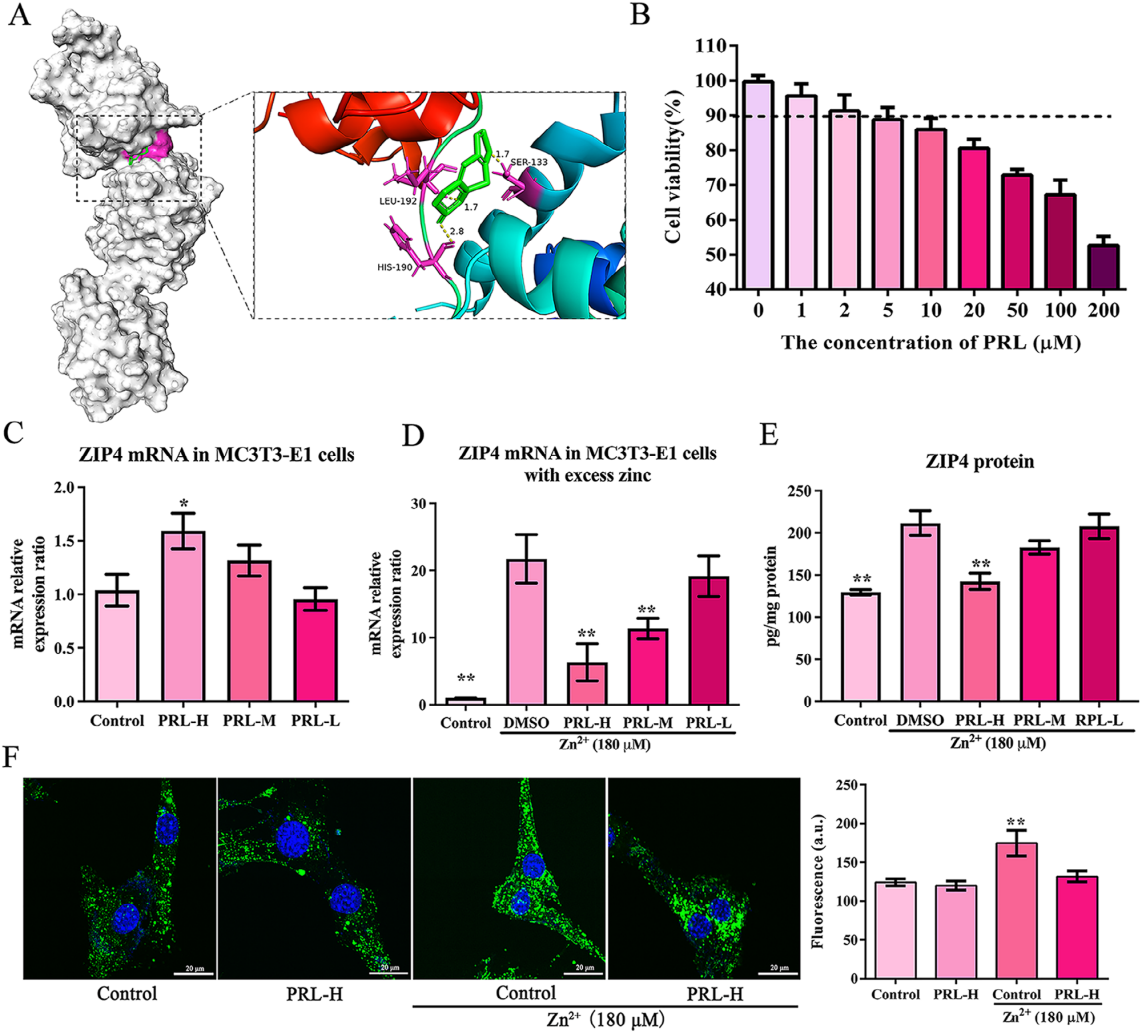
PRL regulates the expression of zinc ion transporter ZIP4. (**A**) Molecular docking of PRL with the protein ZIP4. (**B**) A cell counting kit-8 (CCK-8) assay was used to detect cell viability of MC3T3-E1 treated with different concentrations of PRL (1-200 µM). (**C**) The expression of Zip4 mRNA in MC3T3-E1 cells treated with PRL (1, 2, 5 µM). (**D**) The expression of Zip4 mRNA in MC3T3-E1 cells treated with Zn^2+^(180 µM) for 24 h, then treated with PRL (1, 2, 5 µM). (**E**) The level of ZIP4 protein in MC3T3-E1 cells was determined by ELISA. (**F**) Representative image of FluoZin-3-stained MC3T3-E1 cells, and fluorescence quantification analysis of intracellular Zn^2+^ concentrations. Bars represent mean ± S.D. *p < 0.05; **p < 0.01

To identify drugs that regulate ZIP4 expression, we performed molecular docking analyses of the candidate drugs and the ZIP4 protein. Of these, PRL, the active ingredient in the Chinese herbal medicine *Psoralea corylifolia Linn.*, exhibited suitable steric complementarity with the binding site of ZIP4, with a binding energy of -7.89 kcal/mol. The oxygen atoms of PRL, which are hydrogen bond acceptors, formed hydrogen bonds with the backbone nitrogen atoms of Ser133, His190, and Leu192. Hydrogen bonding interactions also occurred between ZIP4 and PRL (Fig. 2A). We then analyzed the effect of different concentrations of PRL on cell survival rate and selected the doses of PRL to be 1, 2, and 5 µM for subsequent experiments (Fig. 2B). Surprisingly, we found that PRL regulation of ZIP4 is conditional and plays a dual role. Under physiological conditions, PRL slightly up-regulates ZIP4 expression at a dose of 5 µM (Fig. 2C); however, in the presence of excessive Zn^2+^, PRL reversed the upregulation of ZIP4 expression (Fig. 2D-E). Therefore, PRL regulates ZIP4 mRNA expression by balancing the concentration of intracellular Zn^2+^. The results of Zn^2+^ concentration detection showed that PRL maintained the Zn^2+^ in cells at physiological levels (Fig. 2F).

### The characterization evaluation of Zn + CS/pPRL implants

**Fig. 3:**
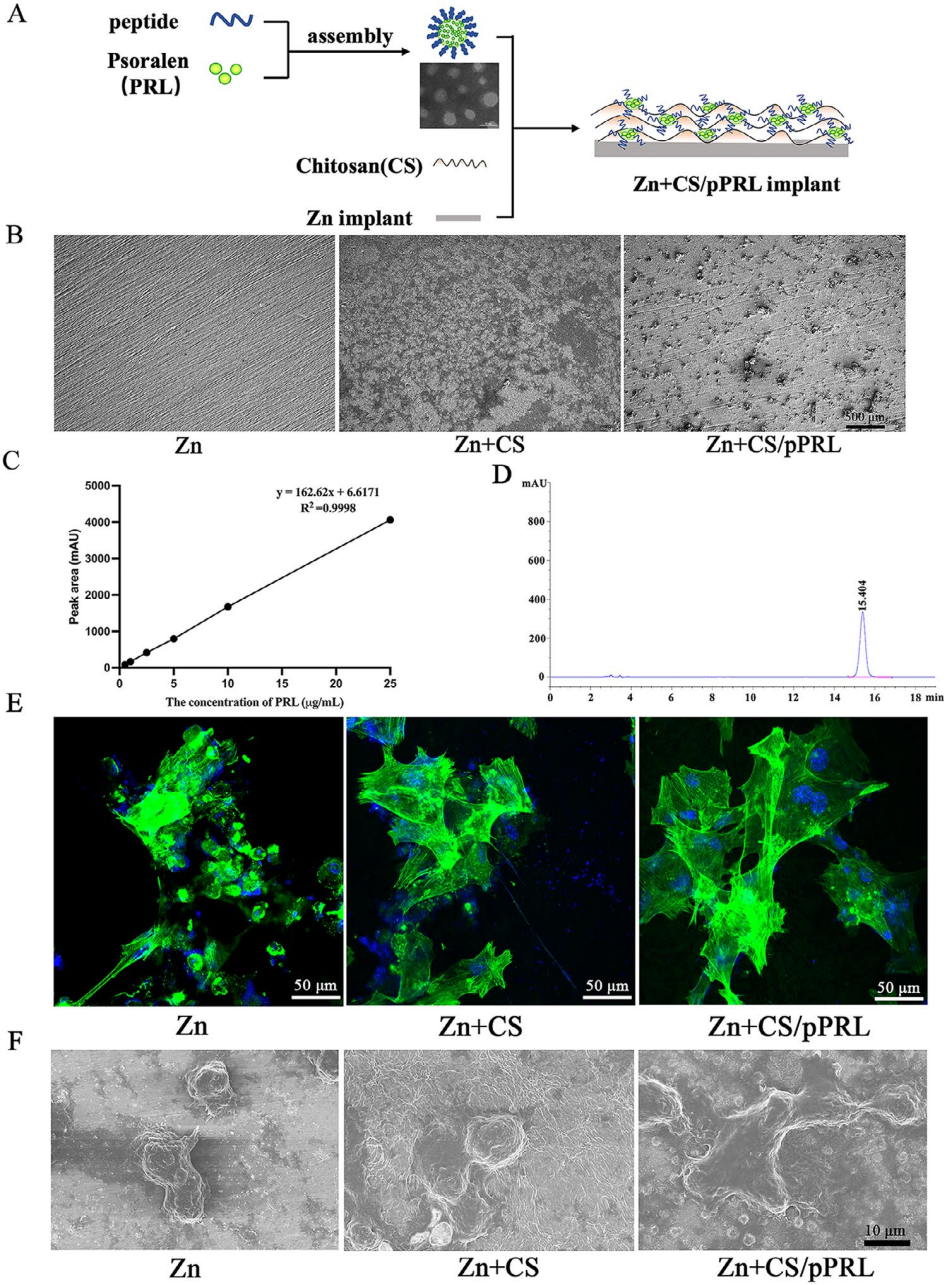
The characterization and cytocompatibility evaluation of Zn + CS/pPRL implants. (**A**) Schematic diagram of PRL loaded on Zn implant surface. (**B**) SEM images of Zn implant surfaces. (**C**) Standard curve for analysis of PRL concentration. (**D**) HPLC chromatograms of PRL concentration analysis. (**E**) MC3T3-E1 cells were cultured on the surface of Zn implants for 24 h, and the cytoskeleton was stained with FITC labeled phalloidin. (**F**) SEM images of cell morphology of MC3T3-E1 cells cultured on the surface of Zn implants for 24 h

Based on the unique ability of PRL to regulate Zn^2+^ concentration, we explored whether PRL loading on the surface of Zn implants improves their cytocompatibility and osteogenic activity. First, we loaded osteogenic targeting peptide-coated PRL (pPRL) onto the surface of the Zn implants via CS (Zn + CS/pPRL) (Fig. 3A), and SEM observation showed that PRL was uniformly loaded on the Zn implants surface (Fig. 3B). We detected about 80 µg/mL of PRL in the Zn + CS/pPRL implant extracts from the HPLC analysis, which indicated that PRL was successfully loaded onto the Zn implants (Fig. 3C-D). Further, cytoskeletal characterization and SEM observation showed that the Zn + CS/pPRL implants showed better cytocompatibility and growth activity than those of the Zn implants (Fig. 3E-F).

**Fig. 4:**
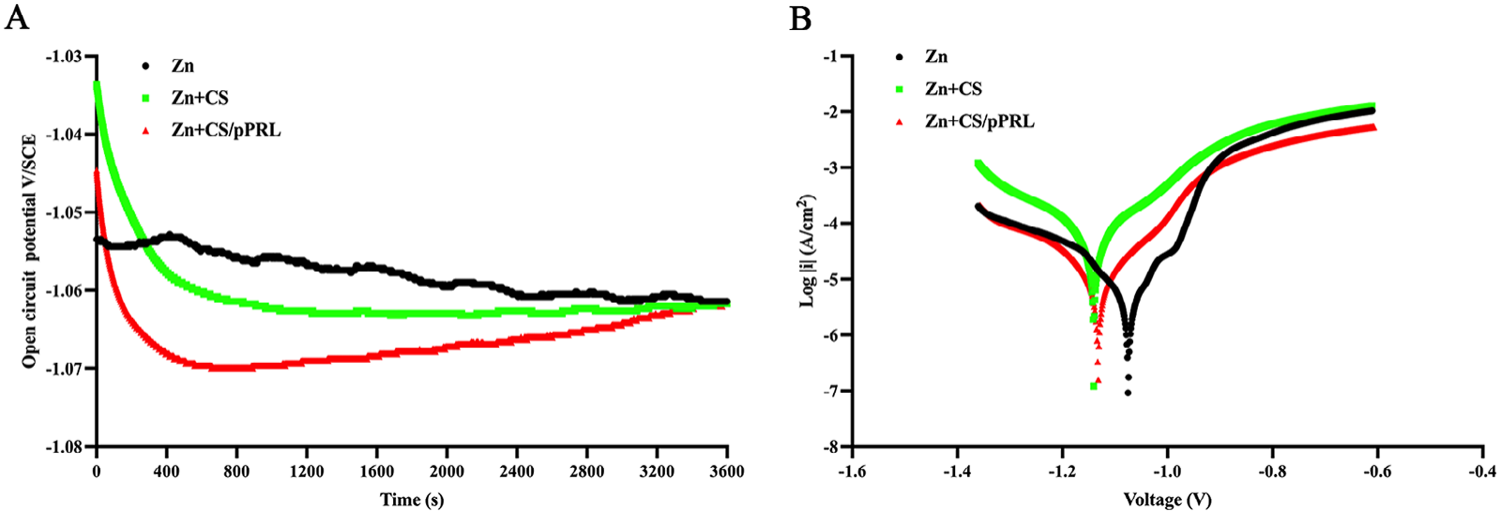
Electrochemical evaluation of Zn, Zn + CS, Zn + CS/pPRL implants. (**A)** Open circuit potential curves. (**B**) Polarization curves of Zn, Zn + CS, and Zn + CS/pPRL implants in SBF

**Fig. 5:**
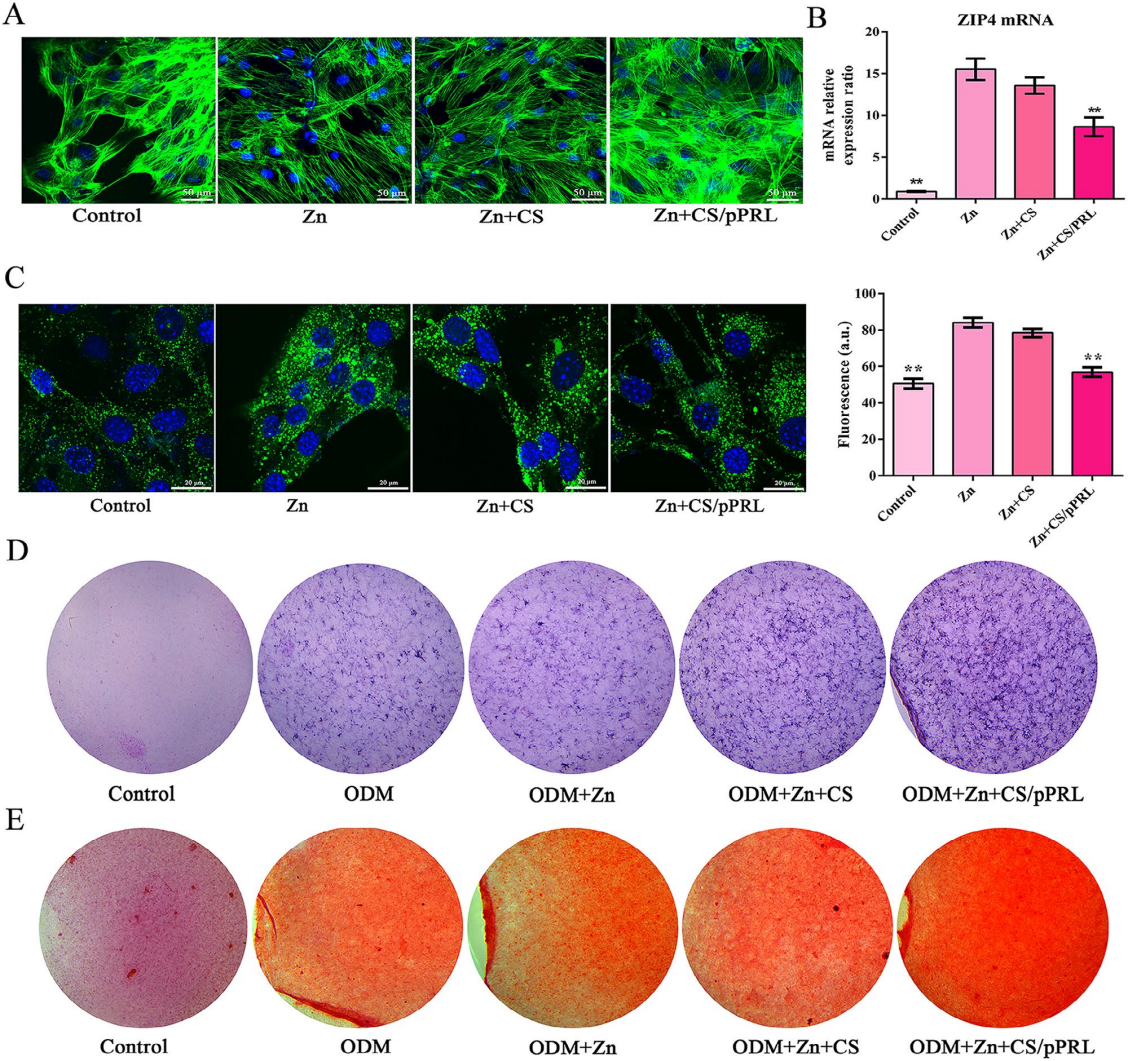
The evaluation of osteogenic activity in Zn + CS/pPRL implants in vitro. (**A**) MC3T3-E1 cells were cultured with 25% extract of Zn implants or Zn + CS/pPRL implants for 24 h, and the cytoskeleton was stained with FITC labeled phalloidin. (**B**) the expression of ZIP4 mRNA in MC3T3-E1 cells treated with 25% extract of Zn implants or Zn + CS/pPRL implants for 48 h. (**C**) Representative image of FluoZin-3-stained MC3T3-E1 cells and fluorescence quantification analysis of intracellular Zn^2+^ concentrations. (**D–E**) MC3T3-E1 cells were cultured with 25% extract of Zn implants loaded with CS or/and PRL for 14 d. The cell activity and mineralization of MC3T3-E1 cells were analyzed using alizarin red S staining and ALP staining. Bars represent mean ± S.D. *p < 0.05; **p < 0.01

Next, the electrochemical behaviors of Zn, Zn + CS, and Zn + CS/pPRL were analyzed using polarization curves. As shown in Fig. 4A, the OCP curve of CS alone or CS/pPRL loaded on the surface of Zn exhibits a rapid decline in the first 1000 s, where it was maintained at -1.061 V_SCE_ until the end, while the OCP curve of pure Zn declined relatively slow. The dissolution and oxygen consumption of the Zn materials were analyzed using the PDP test (Fig. 5B), while the slopes of the anodic and cathodic Tafel changed with surface modification. The corresponding parameters are listed in Table 2. Zn materials formed a narrow passivation zone at -1.065 ~ -0.960 V, and the decomposition potential of this zone increased after CS or CS/pPRL modification of Zn, indicating that the kinetic barrier effect of anodic dissolution is stronger. The Ecorr value and current density on the surface of Zn after CS or CS/pPRL modification were significantly increased, rising to 150.3 and 63.33 µA/cm^2^, respectively (Table 2), which indicates that CS/pPRL-modified Zn surface had a significant influence on the diffusion rate of pure Zn, thereby altering biocompatibility and osteogenic activity of Zn material.

**Table 2:** Electrochemical parameters of Zn, Zn + CS, and Zn + CS/pPRL implants in SBF

Sample	βa (mV/dec)	βc(mV/dec)	Ecorr (V/SCE)	Icorr (µA/cm^2^)
Zn	56.60 ± 11.50	69.23 ± 5.68	-1.049 ± 0.0085	1.753 ± 0.71
Zn + CS	379.30 ± 58.87	296.2 ± 25.01	-1.147 ± 0.0020	150.3 ± 18.55
Zn + CS/pPRL	166.10 ± 3.07	186.0 ± 4.08	-1.126 ± 0.0005	63.33 ± 5.45

### PRL synergizes with Zn implants to promote osteoblast activity

To better evaluate the osteogenic activity of PRL-loaded Zn implants, MC3T3-E1 cells were cultured in a medium containing 25% extract of Zn or Zn + CS/pPRL implants. In the 25% extract of Zn + CS/pPRL implant group, cells manifested greater growth states compared to those with 25% extract of the Zn implant group (Fig. 5A). Analysis of ZIP4 expression in cells revealed that 25% extract of Zn implants induced high expression of ZIP4 mRNA in MC3T3-E1 cells (Fig. 5B), accompanied by an excessively high concentration of intracellular Zn^2+^ (Fig. 5C). Fortunately, in cells incubated with the 25% extract of Zn + CS/pPRL implants, the expression of ZIP4 mRNA decreased (Fig. 5B), and the concentration of intracellular Zn^2+^ was reduced (Fig. 5C). The results of ALP staining and alizarin red staining showed that the 25% extract of Zn + CS/pPRL implants improved the osteogenic activity and mineralization ability of pre-osteoblasts compared with the those of the 25% extract of Zn implants group (Fig. 5D-E).

### PRL modified Zn implants to enhance bone mass in rats with bone defect

**Fig. 6:**
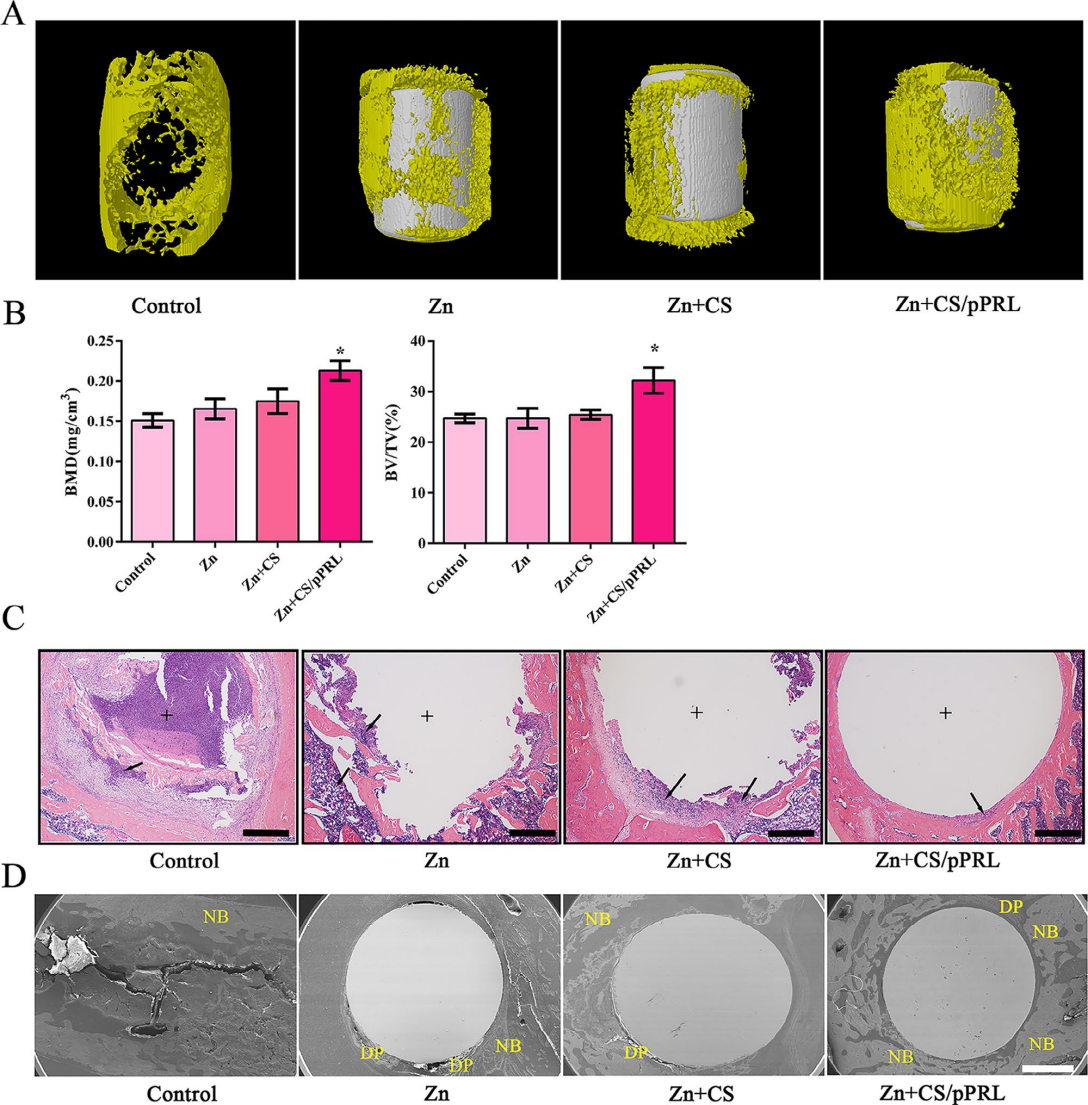
The effects of Zn + CS/pPRL implants on bone mass in rats with bone defect. (**A**) The representative image of Zn implants or Zn + CS/pPRL implants surgically implanted into the femur of rats. (**B**) Representative µCT images showing the 3D bone structures of femurs with bone defects from rats 3 months after Zn implants or Zn + CS/pPRL implants were implanted. (**C**) µCT measurements of BV/TV and Tb.N in femurs with bone defects. (**D**) Representative H&E staining images of femurs with bone defects. Scale bars, 100 μm. (**E**) SEM images of femurs with bone defects. NB: new bone; DP: degradation products; Scale bars, 1 mm. Bars represent mean ± S.D. *p < 0.05

Zn is considered a biodegradable material with great potential for bone formation, and the effect of pure Zn implants or Zn + CS/pPRL implants on bone defect repair was evaluated. A synthetic bone defect larger than 3 mm was created in rats, and the 3 × 4 mm Zn or Zn + CS/pPRL implants were implanted into the bone defect of the rat (Fig. S2). Three months later, bone growth at the bone defect was observed. Bone mass analysis of rat bone defects showed that Zn + CS/pPRL implants had a greater bone mass than that of Zn alone (Fig. 6A-B). Moreover, H&E and SEM analyses showed that more new bone formed around the bone defects in rats with Zn + CS/pPRL implants (Fig. 6C-D) compared to those with Zn implants. In addition, the Zn + CS/pPRL implants did not cause side effects in other organs (Fig. S3).

### PRL modified Zn implants enhance the osteogenic activity by regulating ZIP4

**Fig. 7:**
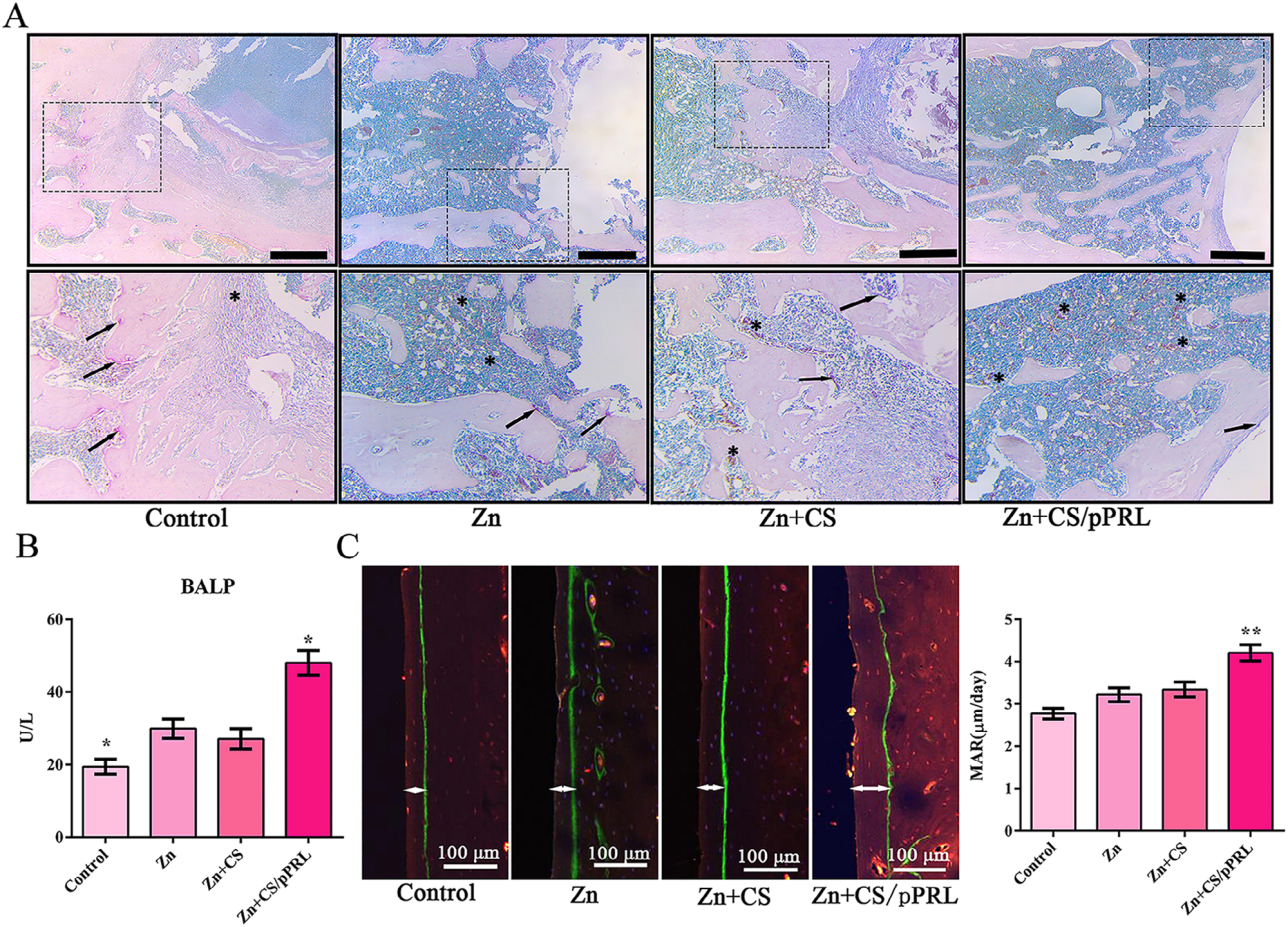
Effects of Zn + CS/pPRL implants on bone formation in rats with bone defects. (**A**) TRAP/ALP staining images of femurs with bone defects from rats. Purple represents TRAP positive; brown represents ALP positive. Scale bars, 100 μm. (**B**) BALP levels in serum detected using an ELISA assay. (**C**) Representative fluorescence images of femurs with bone defects from rats after xylenol orange (red) and calcein labelling (green). Dynamic histomorphometric analyses of femur including mineral apposition rate (MAR). Bars represent mean ± S.D. *p < 0.05; **p < 0.01

**Fig. 8:**
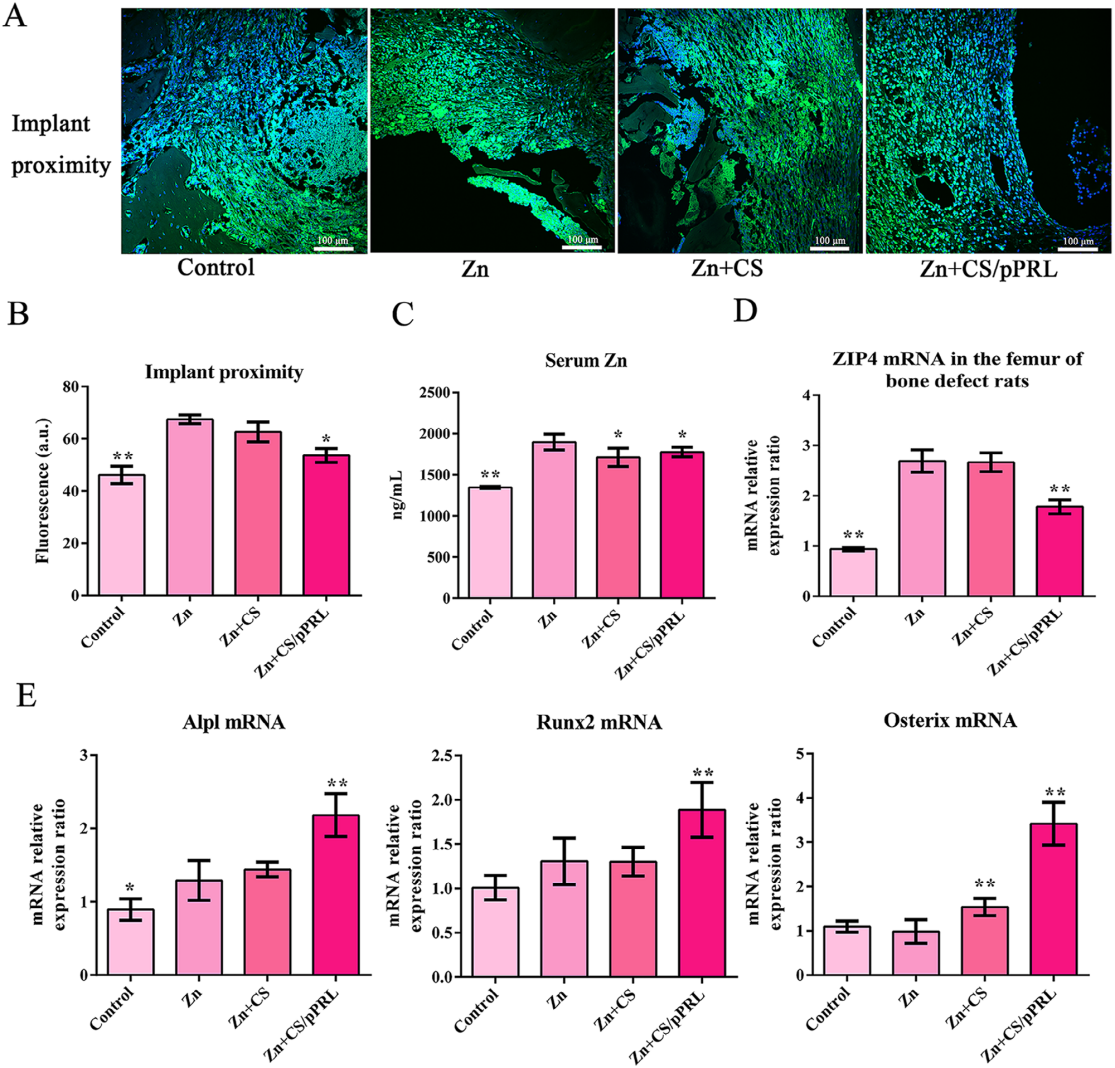
The promotional mechanism of Zn + CS/pPRL implants on bone formation in rats with bone defects. (**A**) Representative image of FluoZin-3-stained femur with bone defects. (**B**)The fluorescence quantification analysis of Zn^2+^ concentrations. (**C**) The serum concentrations of Zn^2+^ detected using ICP. (**D**) The expression of ZIP4 mRNA in femur with bone defects was detected using RT-qPCR (n = 3). (**E**) The expression of Runx2, Alpl, Osterix mRNA in femur with bone defects were detected by RT-qPCR (n = 3). Bars represent mean ± S.D. *p < 0.05; **p < 0.01

Next, we further analyzed the mechanism of Zn + CS/pPRL implants promoting bone repair. TRAP/ALP staining showed that rat femurs with Zn + CS/pPRL implants were more ALP-positive (Fig. 7A). Similarly, the levels of the serum bone formation marker, BALP, significantly increased in rats with Zn + CS/pPRL implants (Fig. 7B). The effect of Zn + CS/pPRL implants on bone formation was evaluated using double-labeling analysis, and the results showed that Zn + CS/pPRL implants accelerated bone formation in rats with bone defects (Fig. 7C). However, this effect was related to the Zn^2+^ concentration and ZIP4 transporter expression. The results showed that the intracellular Zn^2+^ concentrations around the implant and in serum with Zn + CS/pPRL implants was lower than that of rats with only Zn implants (Fig. 8A-C). Finally, ZIP4 mRNA expression was significantly upregulated in Zn-implanted rats compared to that in the Zn + CS/pPRL group (Fig. 8D), while ZIP4 mRNA expression was inhibited in Zn + CS/pPRL implants. Moreover, the expressions of osteogenic genes (Runx2, Alpl, and Osterix) were significantly upregulated in rats treated with Zn + CS/pPRL implants (Fig. 8E), suggesting that PRL, when applied to Zn implants, regulates cellular Zn^2+^ concentrations in vivo by mediating ZIP4 expression and promoting bone repair in rats with bone defects.

## Discussion

Treatment of large bone defects remains a significant challenge for the medical community; thus, many types of implants, especially degradable metal implants, have been developed in vivo. Zn-based biodegradable metal implants have attracted considerable attention as candidates for larger-scale bone-tissue regeneration and support [35]. However, these studies focused on the structure and mechanical support of implants, in fact, side effects caused by degradation explosions and poor biocompatibility restrict their application potential [8, 36, 37], necessitating further research into improving bone regeneration ability. Therefore, to address the problem of cytotoxicity caused by excessive concentrations of Zn^2+^, we searched for related Zn ion transporters and drugs mediating this target to enhance Zn implants, promote bone repair synergistically, and provide a novel candidate, Zn + CS/pPRL, for clinical application in bone repair.

Zinc exists as a divalent cation (Zn^2+^) that is involved in a variety of physiological processes, including signal transduction, apoptosis, and nucleic acid metabolism. Zinc is also required for bone mineralization and wound healing. Zn deficiency is easily observed in stunted growth and loss of appetite; however, Zn toxicity has not been well studied. Zn^2+^ has a biphasic cellular reaction, wherein low concentrations of Zn^2+^ are beneficial to cells while high concentrations (e.g., > 12 ppm) can be significantly harmful [38, 39]. If the extracellular concentration of Zn exceeds Zn homeostasis, an increase in the intracellular Zn concentration triggers apoptosis or DNA damage, followed by necrosis [40]. Murni et al. reported that pure-Zn extract causes necrosis and significant DNA damage in human osteoblast cells [41]. The unresolved issues in the application of Zn in immune repair and the treatment of Alzheimer’s disease are Zn concentration in the plasma and intracellular accumulation, which induce oxidative stress and cytotoxicity [42]. Zn^2+^ excess, as found in our study at concentrations over 360 µM, caused the death of up to 95% of MC3T3-E1 cells, resulting in severe DNA damage. Thus, the physiological importance of Zn homeostasis in humans is particularly prominent, and the surface modification of Zn implants has become a research hotspot to promote biocompatibility and biofunctionalization. However, Zn is required to cross the biological membrane to perform physiological functions; therefore, zinc transporter proteins, especially ZnT and ZIP proteins, are involved in a range of physiological and cellular functions (e.g., skeletal, immune, endocrine, and neuronal functions) wherein they control zinc homeostasis. Gene sequencing analysis revealed that excessive Zn in MC3T3-E1 osteoblasts activated cellular detoxification pathways and inhibited pathways related to osteogenesis and bone growth. This result, owing to the activation of ZIP4 expression, led to an imbalance in Zn levels in MC3T3-E1 osteoblasts and inhibited cell survival and osteogenesis. ZIP4 expression has also been shown to regulate Zn absorption, allowing adequate Zn absorption while also protecting cells from excessive Zn-induced toxicity [43, 44]. Therefore, we believe that maintaining intracellular Zn homeostasis by mediating ZIP4 expression will be helpful in improving the cytocompatibility and osteogenic activity of Zn materials.

PRL, an active component of *Psoralea corylifolia Linn*, has been widely used as a medicinal herb for 1000 years. PRL promotes osteogenesis and bone defect repair by regulating the transcription factors Runx2 and Osterix, which affect osteoblast differentiation and maturation [31], inhibit osteoclast differentiation [45], and regulate extracellular matrix secretion [46]. During the preparation of the Chinese herbal medicine psoralea, the active components of PRL and trace elements of Zn increased [47]. Molecular docking analysis showed that PRL binds to residues Ser133, His190, and Leu192 in the membrane-bound region of ZIP4. Notably, the PRL-mediated ZIP4 expression showed dual characteristics in our study. PRL slightly upregulated ZIP4 expression under physiological conditions; however, PRL reversed the upregulation of ZIP4 expression when Zn^2+^ concentration was excessive. Therefore, PRL-modified Zn materials may improve the cytotoxicity or application limitations caused by excessive local Zn^2+^.

An increasing number of researchers are interested in the application of Zn-based metal implants in bone regeneration and repair because of the need for appropriate Zn ion levels to promote bone mineralization and biological functionalization of bone cell growth and healing [12, 14]. However, during the initial exposure and intensive surface corrosion process, pure zinc implants degrade to zinc phosphate and then zinc oxide in the blood, which affects the local environment and hinders cell attachment by producing highly toxic local Zn^2+^ concentrations. At present, the toxicity of Zn implants is primarily evaluated by incubating cells in extracts. Studies have shown that MC3T3-E1 cells cultured with pure-Zn extract exhibit significant cytotoxicity [8, 48]. Although different amounts of new bone can be formed on bare-Zn-based alloys in vivo, osteoblasts do not perform well when directly incubated with bare-Zn materials [49]. Therefore, reducing the local Zn^2+^ concentration on implant surfaces should improve osteoblast response and accelerate the bone integration of Zn-based implants in vivo. The initial aim of most studies was to inhibit the excessive release of Zn^2+^ and reduce cytotoxicity through a surface stabilization treatment, resulting in a stable surface ZnO film [50, 51]. Conversely, Li et al. showed that ZnO films, formed by autoclaving Zn implant surfaces exhibited significant cytotoxic effects on fibroblasts [52]. Moreover, some studies have shown that surface modification methods, such as micro-arc oxidation or poly(l-lactic acid) (PLLA)-modification of pure Zn, increase the local concentration of Zn ions, leading to more significant cytotoxicity [53, 54]. Thus, it is controversial that surface anti-corrosion treatment of Zn materials reduces the release of zinc to improve their cytocompatibility. In the present study, Zn implants were coated with PRL encased in nanoparticles formed from amphiphilic peptide molecules. The results showed that PRL could affect the cytocompatibility of Zn implants by controlling intracellular Zn ion concentrations.

In terms of clinical requirements, bone-repair implants should have significant osteogenic activity. Bioactive coatings prepared on implant surfaces have been extensively studied to significantly improve tissue integration [55]. For example, Zn-based scaffolds containing the growth factor IL-4 [56] are used as bone graft substitutes for segmental or large bone defects. However, there is a lack of research on drug-modified Zn implants for enhancing the osteogenic activity. In vivo studies have shown that PRL-modified Zn implants significantly promote bone formation and accelerate bone-defect repair in rats. Drug-loaded coatings, such as paclitaxel [57], sirolimus [58], and phosphorylcholine [59], reportedly improve the antihyperplasia and hemocompatibility of Zn-based materials. Therefore, Zn implants loaded with drugs can enhance their pharmacodynamic activity and expand their clinical application.

## Conclusions

In this study, we identified ZIP4, a Zn transporter protein, as a novel target for inhibiting excessive Zn^2+^-induced cytotoxicity to better understand and refine the physiological functions and toxic effects of Zn^2+^ in vivo. The natural compound PRL was used to regulate the expression of ZIP4 and redistribute the content of intracellular Zn^2+^ to reduce the cytotoxicity caused by excessive local Zn^2+^ and promote bone formation, thereby promoting bone repair in rats. These findings provide new insights for improving the cytocompatibility of Zn-based implants and accelerating bone repair for larger scale bone defects.

### Electronic supplementary material

Below is the link to the electronic supplementary material.

**Supplementary Figure S1.** (A) MC3T3-E1 cells were transfected with siRNA-Control or siRNA-ZIP4 for 24 h, and then incubated with cell medium containing excessive Zn^2+^ (180 µM) or control for 24 h, and cell counting kit-8 (CCK-8) assay was used to detect cell viability. (B) Apoptotic cells were detected using a Terminal dUTP nick-end labeling (TUNEL) assay. (C) The quantification analysis of positive TUNEL cells. Bars represent mean ± S.D. *, *p* < 0.05; **, *p* < 0.01

**Supplementary Figure S2.** A surgical image of pure Zn, Zn + CS, or Zn + CS/pPRL implants implanted into the rat with bone defect

**Supplementary Figure S3.** Representative images of heart, liver, spleen, and kidney stained by H&E.

## Data Availability

Data will be made available on request.

## Abbreviations

BALP: Bone alkaline phosphatase
CS: Chitosan
FBS: Fetal bovine serum
FITC: Fluorescein isothiocyanate
HPLC: high-performance liquid chromatography
PINP: Procollagen type I N-terminal propeptide
PRL: Psoralen
PBS: Phosphate-buffered saline
TEM: Transmission electron microscopy
SEM: scanning electron microscopy
Zn: zinc
Zn^2+^: zinc ion
ZIP4: Zrt- and Irt-like protein 4
