# Supplementary figures and images for: Psoralen synergies with zinc implants to promote bone repair by regulating ZIP4 in rats with bone defect
Source: Biomater Res. 2023 Dec 10;27:129. doi: 10.1186/s40824-023-00472-w (PMC10712184; doi:10.1186/s40824-023-00472-w)

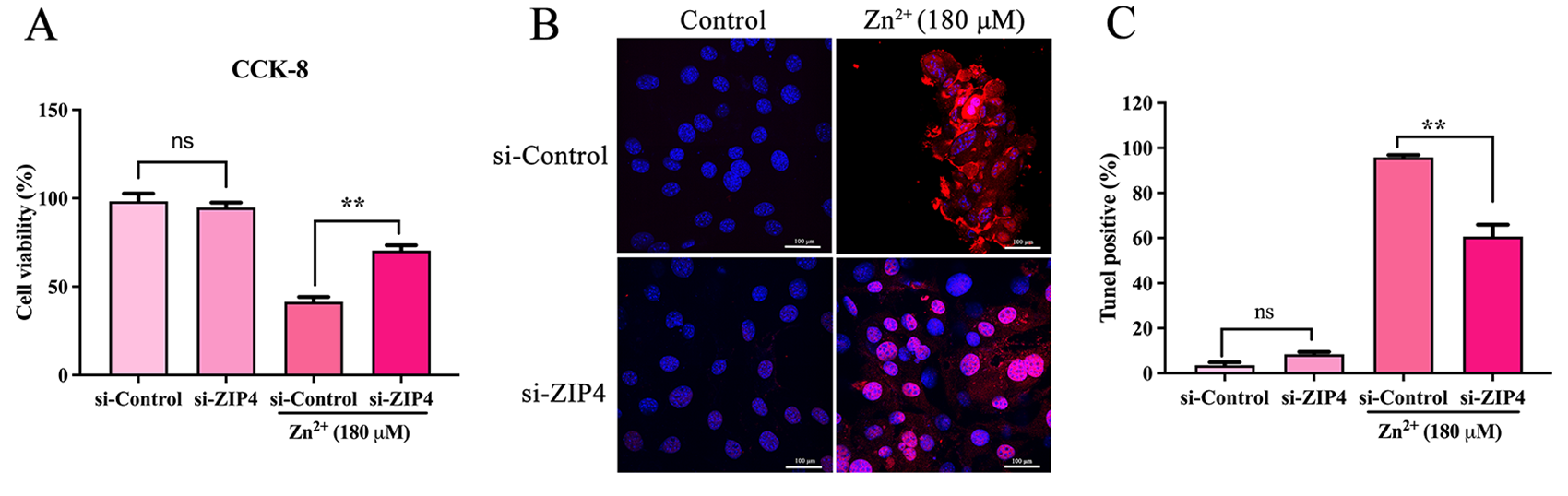

Supplement: Supplementary file 1 — Supplementary Figure S1. (A) MC3T3-E1 cells were transfected with siRNA-Control or siRNA-ZIP4 for 24 h, and then incubated with cell medium containing excessive Zn2+ (180 µM) or control for 24 h, and cell counting kit-8 (CCK-8) assay was used to detect cell viability. (B) Apoptotic cells were detected using a Terminal dUTP nick-end labeling (TUNEL) assay. (C) The quantification analysis of positive TUNEL cells. Bars represent mean ± S.D. *, p < 0.05; **, p < 0.01 [file 40824_2023_472_MOESM1_ESM.tif]

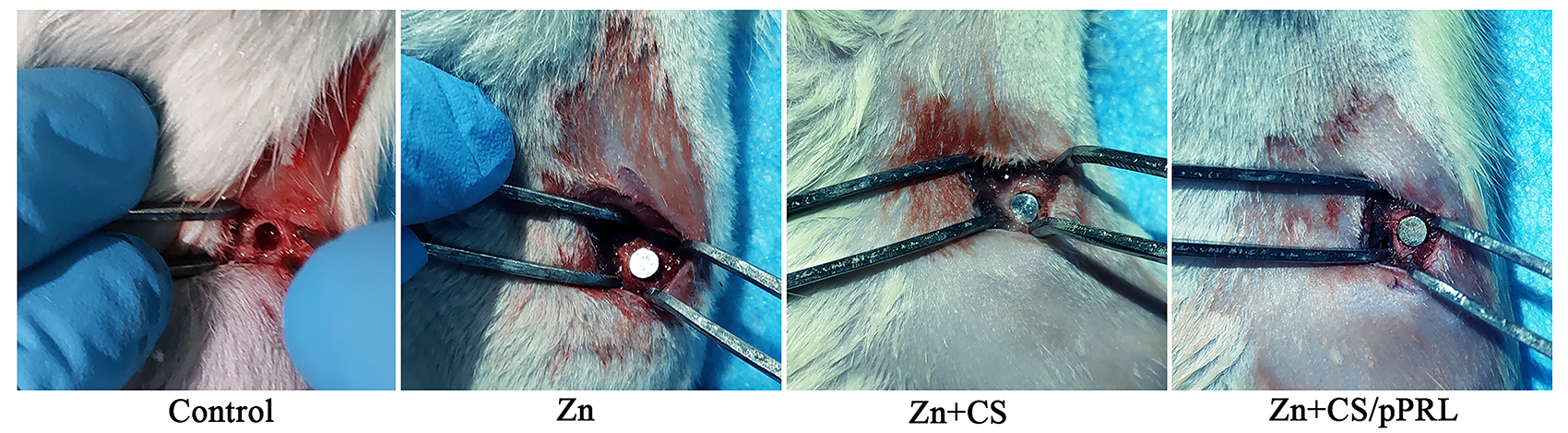

Supplement: Supplementary file 2 — Supplementary Figure S2. A surgical image of pure Zn, Zn + CS, or Zn + CS/pPRL implants implanted into the rat with bone defect [file 40824_2023_472_MOESM2_ESM.tif]

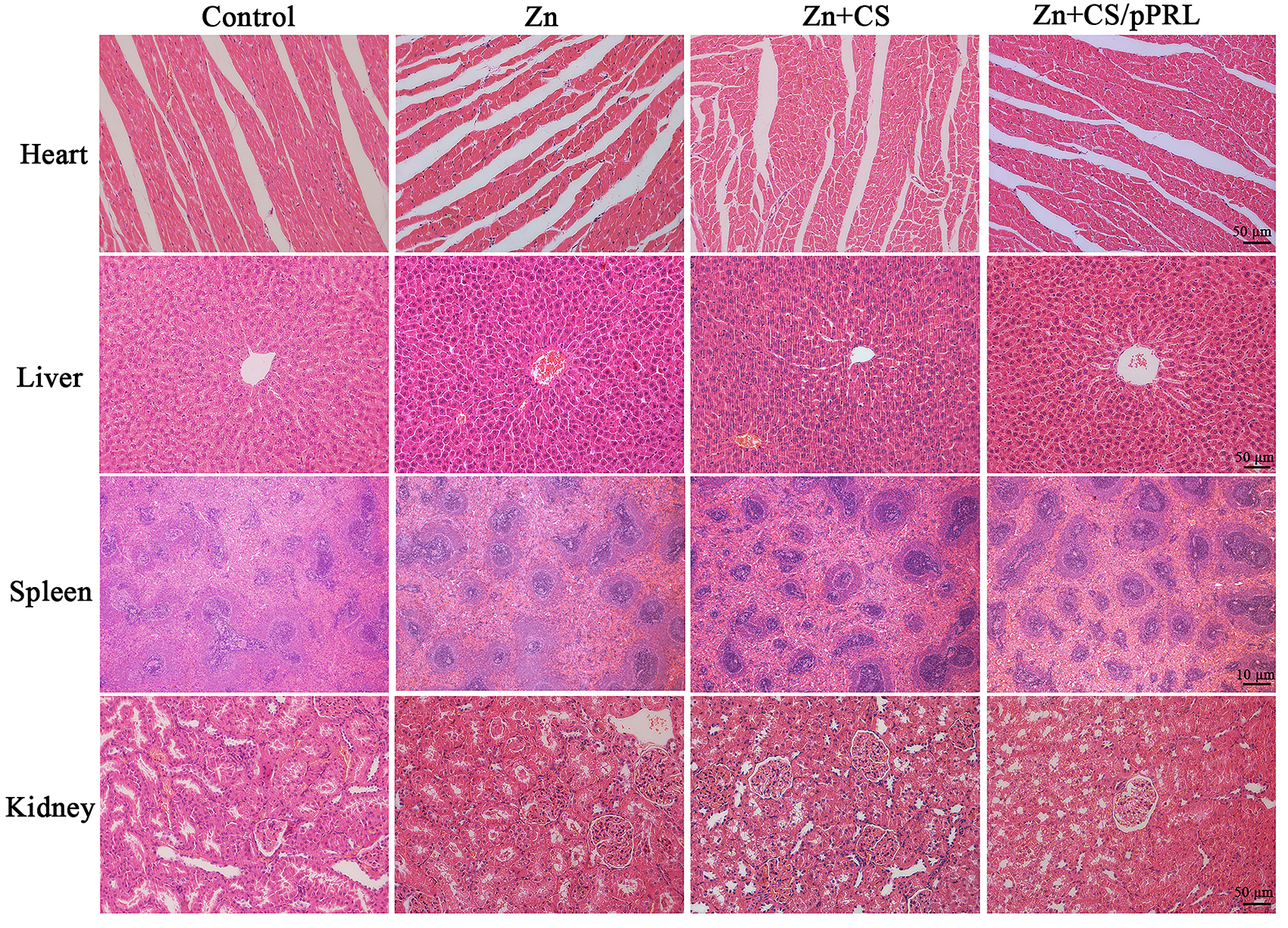

Supplement: Supplementary file 3 — Supplementary Figure S3. Representative images of heart, liver, spleen, and kidney stained by H&E. [file 40824_2023_472_MOESM3_ESM.tif]
